# An introduction to Delphi studies and consensus methods for genetic counselors

**DOI:** 10.1002/jgc4.70136

**Published:** 2025-11-17

**Authors:** Ian M. MacFarlane, Heather Zierhut

**Affiliations:** ^1^ Department of Genetics, Cell Biology, and Development University of Minnesota – Twin Cities Minneapolis Minnesota USA

**Keywords:** consensus methods, Delphi, policy, research, statistics

## Abstract

Delphi studies, a type of consensus method, are instrumental in healthcare research for gathering expert perspectives, especially when conclusive evidence is not available. Developed in the 1950s, Delphi methodology is characterized by anonymity, iteration, controlled feedback, and statistical group response. The traditional Delphi method, along with its subforms, policy and decision, has been widely used across various fields, including genetic counseling. In genetic counseling, Delphi studies have been used for guideline development, curriculum design, clinical competency selection, and establishing quality metrics. The overall goal of this research methodology article is to explain the potential benefit of using a Delphi method in the field of genetic counseling and differentiate the Delphi method from other consensus methods available. Educational applications include creating curricula for Master's programs and defining competencies for clinical supervision. Delphi studies have also been used to develop core outcome sets and standardize outcome reporting measures in genetic counseling research. Quality assessment in genetic services has also been studied using Delphi studies. In addition to summarizing Delphi studies in genetic counseling, we provide an overview of the major questions to consider when constructing a Delphi protocol. We discuss common design and provide practical tips for implementation such as: who counts as an expert, how to decide how many rounds to do, how to set up the questionnaire, and how to report findings of a Delphi study. Researchers should thoughtfully consider these many points and the impacts these choices may have on their overall study results.

**What is known about this topic**

Consensus methodology broadly, and the Delphi method specifically, are useful tools to explore research questions where data to answer the question directly are lacking. Delphi methodology uses iterative interactions with experts using surveys to arrive at a consensus opinion. Delphi methodology is complex and the details of implementation vary widely in the literature.

**What this paper adds to the topic**

This article provides an overview of the Delphi method, with a brief review of other consensus models for context. We provide useful background information as well as guidance on key decisions for the design, analysis, and reporting processes to facilitate more rigorous research by genetic counselors when using this approach.

## 1 INTRODUCTION

Delphi studies belong to a class of designs called consensus methods, which collectively seek to gather the perspectives of experts to answer a research question. In healthcare, consensus methods are often useful for situations where there is insufficient evidence from the research base to make a decision based on outcome data alone (Campbell & Cantrill, 2001). Consensus methods can therefore be utilized as part of the decision‐making process to systematically gather expert opinion to supplement available data (Murphy et al., 1998). Consensus methods arose to improve the likelihood of groups to work toward solutions based on the strength of ideas rather than other interpersonal factors (e.g., more junior members of a group may defer to those with more experience or a more confident person may convince others to accept their idea even with flaws).

Delphi was the first of these consensus methods. Since being developed in the 1950s, Delphi methodology has been used in many fields and adapted in numerous ways (Crisp et al., 1997). There is some agreement about a set of four core characteristics of a Delphi study (e.g., Rowe et al., 1991):

1. Anonymity: No direct communication occurs between panelists (the participants in the study), who are anonymous to each other. Data are collected exclusively by survey (originally via mail but increasingly online). This is meant to eliminate factors, such as prestige, social dominance, and subgroups forming. Anonymity also serves to empower panelists to put forward ideas that might be unpopular and change their minds without losing face.
2. Iteration: Repeated presentation of the evaluation survey in a structured manner provides opportunities for panelists to change their minds at regular intervals.
3. Controlled feedback: Between rounds of the survey, the researchers analyze the data provided by the panelists and create written summaries that are distributed to panelists with the next iteration of the survey. The nature of the feedback varies, but is most often given as descriptive statistics and/or comments from the panel explaining their rationale. This structured feedback ensures all panelists' inputs contribute to the next round of deliberation.
4. Statistical group response: Once consensus is reached, the final product is a group judgment expressed with descriptive statistics. Most often the statistics provided include a measure of center (e.g., mean, median) to indicate the experts' position alongside a measure of spread (e.g., standard deviation, interquartile range, range of values) to convey the degree of consensus.

The traditional Delphi method, often referred to as either classic Delphi or just Delphi, is the most common form but there are several other widely recognized subforms of Delphi in the literature. The policy Delphi subform seeks to first create a broad list of options before developing consensus from those most directly affected by a policy under consideration (Rayens & Hahn, 2000). A decision Delphi is even more specific with the goal of determining how to move forward with a specific choice among those with decision‐making power (Rauch, 1979). Additional subforms continue to be developed (e.g., Niederberger et al., 2024, lists eight subforms) but at their core, Delphi studies gather input from different experts and synthesize that expertise into usable data for many purposes.

The objective of this article is to provide researchers with a brief overview of Delphi methods as a starting point for developing genetic counseling studies utilizing these methods. With the four core criteria of a Delphi study explained, we will highlight the ways genetic counseling researchers have used Delphi to illustrate the variety of potential applications. Next, we will distinguish Delphi methods from similar consensus‐building approaches, followed by overviews of the primary decision points in designing and executing a Delphi study. We conclude with limitations of Delphi approaches. We encourage researchers who plan to use this methodology to review the sources we provide for more depth and nuance, as many aspects of Delphi methods are still under debate in the literature.

### 1.1 Delphi method applications in genetic counseling practice and research

Genetic counselors often face situations in patient care or variant curation where evidence is contradictory, incomplete, or absent, and thus have to rely on their clinical experience and/or judgment. Consensus methods broadly, and Delphi approaches specifically, are therefore a promising tool for genetic counseling research to inform practice. Delphi studies in genetic counseling have already been employed for a variety of reasons including the development of clinical guidelines, the design of educational curricula, the identification of essential clinical competencies, and the establishment of core outcome sets and quality metrics for genetic service delivery (see Table 1). One of the most prominent and highly impactful uses is to reach expert consensus to inform clinical guidelines (Murphy et al., 1998). Consensus methods help ensure that clinical guideline recommendations are both evidence‐informed and contextually relevant. A notable genetic counseling example is the creation of recommendations for integrating genetic counseling services into cystic fibrosis care centers (Langfelder‐Schwind et al., 2022). A Delphi study was also used to develop clinical genetic counseling and testing guidelines for amyotrophic lateral sclerosis in the United States, providing a framework for navigating complex ethical, diagnostic, and familial considerations in neurogenetic care (Roggenbuck et al., 2023). Educational‐related Delphi studies include the development of curriculum for Master's programs in Europe and the creation of a list of competencies for clinical supervision in genetic counseling (Eubanks Higgins et al., 2013; Skirton et al., 2013). Other consensus lists of core competencies have been developed for nongenetics healthcare professionals including one tailored to the specific clinical domain of cancer genomics (Hoxhaj et al., 2022; Tognetto et al., 2019).

**TABLE 1:** Examples of Delphi and other consensus‐building methodologies in genetic counseling research.

Study	Purpose	Participants	Panel size	Rounds	Stopping rules
Borle et al. (2022)	Assess perspectives on if changes in service delivery and genomic testing would be implemented by 2030	Canadian genetics professionals	27	2	Stability: significant difference in scores between rounds Consensus: >75% selecting the same Likert score or within 10% on sliders
Chou et al. (2019)	Develop metrics for quality assessment for genetic services	US experts in genetics and/or performance measurement	24	4	Not reported; “reached consensus”
Eubanks Higgins et al. (2013)	Identity genetic counseling supervisor competencies	Program directors, assistant program directors, and experienced supervisors from ABGC accredited genetic counseling programs	74	2	Not reported; observed “strong agreement”
Jacobs et al. (2017)	Determine if messages received by women with breast/ovarian cancer who undergo BRCA 1/BRCA 2 testing are key or not key	Expert oncology/genetics health professionals and patients with breast/ovarian cancer and BRCA 1/BRCA2 pathogenic variant	32	3	> 75% agreement
Kohler et al. (2017)	Determine whether genomic sequencing outcomes were considered as having personal utility to patients	ClinSeq Project study participants	40	2	“…small number of changes in responses to plausibility of the items and the drop off rate of the respondents”
Langfelder‐Schwind et al. (2022)	Determine the strategies and standards of care needed to remedy disparities in genetic counseling access for parents of CFNBS+ infants	CF physicians, GCs, NBS lab directors and researchers, parents of children with CF	17	3	>80% agreement to accept statement as written
Michie et al. (1998)	Assess consensus about the objectives of genetic counseling	Public health doctors, regional advisors in general practice, clinical geneticists, GCs, out‐patients	115	2	Not reported
Paneque et al. (2015)	Identify aspects of effective counseling practice in presymptomatic testing for late‐onset neurological disorders	European medical doctors, geneticists, genetic counselors, and genetic nurses	29	3	Not reported
Redlinger‐Grosse et al. (2021)	Assess stakeholders' priorities for genetic counseling outcomes	Clinical GCs, outcome researchers, genetic counseling training directors, genetic counseling consumers/advocates	52	2	Not reported
Roggenbuck et al. (2023)	Develop clinical ALS genetic counseling and testing guidelines	Academic neurologists, community neurologists, genetic counselors, physician scientists, laboratory experts, ALS advocates, and people with ALS	Not reported	3	>80% agreement on yes/no evaluation of statement
Skirton et al. (2013)	Develop a core curriculum for genetic counselors in Europe	Experienced expert genetic counseling practitioners and/or educators working in Europe	35	2	>70% of participants rate item as “essential”

Abbreviations: GC, genetic counselor; CF, cystic fibrosis; NBS, newborn screening.

Delphi methodologies have also been effectively employed to evaluate and enhance the quality of care within genetic services. These types of studies help to identify and prioritize tools and metrics to measure outcomes of service delivery. For example, Chou et al. (2019) utilized a Delphi process to select an appropriate quality assessment tool for genetic services, ultimately identifying 16 candidate tools categorized into five key domains: service capacity, access to care, data systems, performance reporting, and patient‐centered outcomes. In another application, Paneque et al. (2015) conducted a Delphi study with European experts to define effective genetic counseling practices for individuals with late‐onset neurological disorders, a context that presents unique ethical and psychosocial challenges. Similarly, Jacobs et al. (2017) used a Delphi approach to assess and refine key messages for communicating genetic cancer risk information to women diagnosed with breast or ovarian cancer in the United Kingdom, emphasizing the importance of clarity, empathy, and informed decision‐making in patient communication.

Beyond quality assessment and communication strategies, Delphi studies have contributed to the development of core outcome sets (COS) for genetic counseling and testing research. COS are standardized sets of outcomes that should be measured and reported in all studies within a specific field, promoting consistency and comparability across research efforts. Redlinger‐Grosse et al. (2021) applied a Delphi process to prioritize outcomes in genetic counseling, beginning with a comprehensive list of 181 potential outcomes. These were rated by experts based on perceived importance and subsequently organized using the Framework for Outcomes of Clinical Communication Services in Genetic Counseling. This work represents a critical step toward harmonizing outcome reporting in genetic counseling research and enhancing the evidence base for clinical practice to get closer to a recognized COS for genetic counseling.

Delphi studies have been employed to explore emerging and conceptual dimensions of genetic counseling practice, offering a structured means of capturing diverse stakeholder perspectives. One such application involved the prioritization of genetic counseling objectives from the viewpoints of consumers, genetic counseling providers, and referring physicians in the United Kingdom. This early study by Michie et al. (1998) highlighted the value of triangulating perspectives to inform patient‐centered care and service delivery priorities. More recently, Kohler et al. (2017) used a Delphi process to define the concept of personal utility in the context of individual genome sequencing results. This work contributed to a broader understanding of how individuals derive meaning and value from genomic information beyond clinical outcomes, such as through psychological, social, or behavioral impacts. In another forward‐looking application, Borle et al. (2022) conducted a Delphi study to gather expert opinions on the future of clinical genetic and genomic services in Canada. This study provided insights into anticipated challenges and opportunities in service delivery, workforce development, and integration of genomic technologies into mainstream healthcare. Collectively, these studies demonstrate the versatility of the Delphi method in addressing both practical and conceptual questions in genetic counseling, from defining service goals and patient values to forecasting the future of the profession.

### 1.2 Alternative consensus methods

With all these examples of Delphis in genetic counseling research as a base, it is also important to distinguish the Delphi approach from other consensus‐building methodologies that share some common elements. The nominal group technique (NGT; Delbecq & Van de Ven, 1971), is similar to a Delphi in that it uses an iterative process of estimation and feedback. Originally developed as a process for committee decision‐making, NGT differs from Delphis by seeking to limit domineering personalities and other interpersonal dynamics through highly structured interactions and the separation of idea generation from discussion rather than anonymity (Murphy et al., 1998). The panel (usually 9–12) meets together, typically in person (though sometimes the initial step of idea generation is done remotely), with a trained facilitator who has subject matter and/or process expertise (Jones & Hunter, 1995). Background information is often, though not always, presented to panelists for review (Humphrey‐Murto et al., 2017). The panelists are first asked to individually write down all their ideas relevant to the topic. After the writing period, each member is asked to read aloud the most important idea from their list that has not yet been shared, followed by the next person's most important new idea, and so on until all ideas have been contributed. Each idea is then discussed by the panel, after which the members individually rank order the ideas based on a specified characteristic (e.g., importance, feasibility). The ranks are cumulatively tabulated and numerical feedback is given to the panel. The panel then continues to discuss the ideas followed by ranking and feedback until consensus is reached. The advantages of NGT are the ability for panelists to discuss the ideas directly, ask for clarification, and/or provide additional information to support their position (Humphrey‐Murto et al., 2017). The drawback is the interpersonal pressures the Delphi seeks to avoid (e.g., deference to prestige, domineering personalities) may still occur, largely dependent on the skill of the facilitator and the willingness of panel members to interact according to the rules of the process.

Another consensus method is the RAND/UCLA Appropriateness Method (RAM; Fitch et al., 2001) which was developed specifically in healthcare to assess whether procedures would be appropriate for patients based on different combinations of symptomology, medical history, and lab results. A detailed literature review ascertains the most up‐to‐date evidence about the procedure and is used to develop clinical scenarios. These scenarios often include components like patient demographics, presenting concerns, medical history, test results, and/or provider decisions. Key variables are often different across scenarios to assess the impact of these factors on decision‐making (e.g., in Scenario 1 there is a known family variant while in Scenario 2 there is not). The background and scenarios are sent to a group of experts who independently rate each scenario based on expected harm‐to‐benefit ratio on a scale of 1–9. The panelists are then brought to a 1–2 day meeting where a facilitator presents the results of the first round and leads discussion that can result in the modification of scenarios and/or definitions. The panelists then re‐rate each scenario individually. The procedure is ultimately rated as appropriate, inappropriate, or uncertain based on median ratings and degree of consensus. The goal of the RAM is not explicitly to reach consensus. Instead, the process is meant to differentiate between difference of opinion based on clinical judgment from disagreement based on misunderstanding, lack of clarity, or fatigue with the process. An optional third round may take place if necessity must also be assessed alongside appropriateness, though this round is typically done remotely.

Consensus conferences were also developed specifically for healthcare by the National Institute of Health, merging aspects of a judicial trial, a scientific conference, and a town hall meeting (Jacoby, 1985). These conferences typically occur over several days, starting with presentations of evidence by experts, including discussion with the panel. Both of these portions are conducted in a public forum where the audience is encouraged to participate by asking questions or sharing opinions. The panel then shifts to a closed deliberation to produce a consensus statement. Many nations have adopted similar processes (e.g., McGlynn et al., 1990) and consensus conferences have also been used outside the healthcare context (e.g., agriculture; Porsborg Nielsen et al., 2007).

Given the variety of consensus methods available, genetic counseling researchers should think carefully about the goals of their study and practical considerations (e.g., access to experts, funding, timeframes) when selecting what approach will work best. A full exploration of the nuances between approaches is beyond the scope of this article, but the references cited will provide a gateway to digging deeper. As Delphi methods allow for asynchronous participation of expert panelists and remove the costs associated with travel, this approach seems most accessible to genetic counseling researchers and has already been used with success in our field. The remainder of the article will focus on the major decisions to make when designing, executing, and reporting studies using the Delphi method, followed by limitations of the Delphi approach.

## 2 DESIGNING A DELPHI STUDY

**FIGURE 1:**
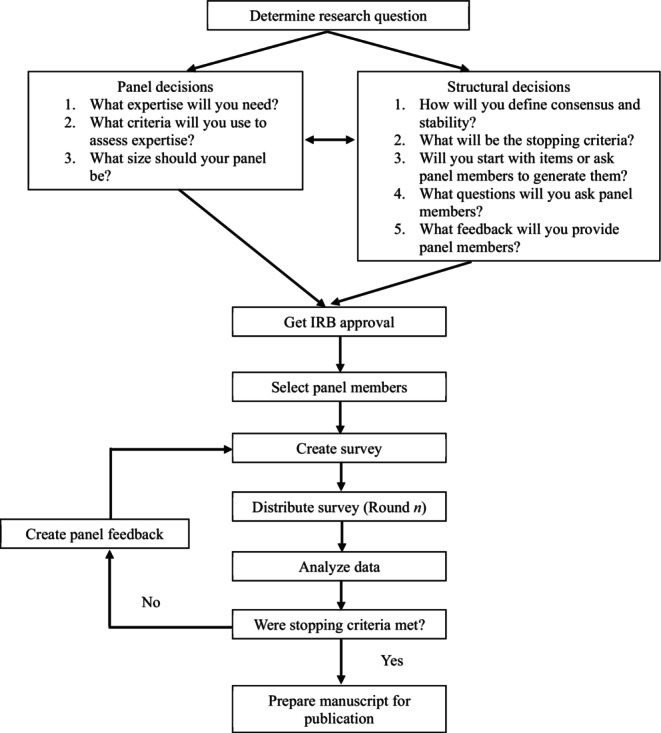
Steps in executing a Delphi study.

In this section, we provide an overview of the major decision points when constructing a Delphi protocol (see Figure 1 for overview). At each stage, we discuss common options along with their pros and cons, share identified criticisms, and provide some practical tips for implementation.

### 2.1 Who counts as an expert and how many are needed?

Once the research question is set, it is time to determine who will be the experts for the panel. Several authors describe this step as the most important in the study design, as the credibility of the study only goes so far as the experts are trusted (e.g., Hsu & Sandford, 2007; Nasa et al., 2021). The definition of “expert” is not well defined in the Delphi literature (Diamond et al., 2014; Nasa et al., 2021), so the context of the research question should determine the criteria for expertise. Common criteria include prestige/renown, organizational affiliation, relevant experience, and recommendations/nominations (Boulkedid et al., 2011; Junger et al., 2017). While these criteria have the advantage of being relatively easy to identify, count, and rank, researchers have to be cognizant of the power structures within healthcare and education that have systematically limited access and upward career mobility based on identity factors, such as race, gender, sexual orientation, ability status, religion, socioeconomic status, and many other intersectional components. Researchers are likely to reify these power structures without careful consideration of how to determine “expert” status. Determination of whose perspectives are present or absent in the panel is an important consideration for questions related to healthcare due to the complex interplay of physical and social determinants of health. Researchers must be careful, however, to avoid tokenization of group perspectives, such as assuming having one panel member with a disability ensures the “disabled perspective” has been addressed. Spickermann et al. (2014) encourage researchers to consider both surface‐ (e.g., identity‐based) and deep‐level (e.g., values, skills) factors when assessing panel composition.

There may be multiple types of expertise needed to address the research question holistically, so criteria for selection may vary across subtypes of experts. What makes someone an expert clinician is likely quite different from expertise in policy analysis, and both are different from lived experience with a genetic condition. At times it may even be helpful to analyze data from these subgroups of experts separately. For example, Redlinger‐Grosse et al. (2021) used a Delphi method to explore prioritization of genetic counseling outcomes and compared results across clinical genetic counselors, program directors, researchers, and consumers/advocates.

Once the criteria have been established, the process of identifying experts begins. Okoli and Pawlowski (2004) suggest the use of a knowledge resource nomination worksheet (KRNW) to help organize the search. This tool helps identify disciplines, skills, organizations, and literature where expertise may be found. Using this framework, researchers can start listing names of potential experts from these categories. Once the initial list is generated, contacting those experts and asking them to nominate additional experts is recommended. Whether one uses the KRNW formally or not, this process of snowball sampling recommendations is encouraged in the literature (e.g., Fink‐Hafner et al., 2019; Hsu & Sandford, 2007; Sablatzky, 2022).

The target size of the panel is another important consideration. Depending on how expertise has been defined, the pool of potential experts could be quite small or enormous. The typical panel is between 10 and 100 for most Delphis (e.g., Fink‐Hafner et al., 2019; Nasa et al., 2021), though reviews in healthcare fields have found panels ranging from 3 to >1500 (e.g., Boulkedid et al., 2011; Zartha Sossa et al., 2019). While most quantitative study designs use power analyses to determine the required sample size, the descriptive nature of the analyses typical of Delphi studies (discussed further below) precludes the need for inferential statistics, making power moot. Researchers generally maximize their samples to increase precision, but the potential for attrition must also be considered given the multiround nature of Delphis. When experts drop out, it is unclear if consensus improving in subsequent rounds is due to true consensus building or those with differing opinions are leaving the study. Attrition is problematic for any study, but the relatively small panel size of Delphis makes this particularly challenging.

Depending on the desired size of the potential pool of experts, snowballing could be repeated until no new names are provided or until the predefined target size is reached. The next step is to rank the finalized list of potential candidates by the criteria previously established. This could be one overarching list, but it may be more useful to create sublists based on categories of expertise. Experts are then invited in order of their ranking, either overall or by category, until the target panel size is reached. About 6% of studies in Foth et al.'s (2016) review randomly selected their experts, so this is another option.

### 2.2 How many rounds are needed?

Consensus methodologies are based on the assumption that a variety of opinions currently exist about the research question. If a well‐established and nearly universal perspective already exists, there is no need for consensus building (e.g., we do not need a Delphi to establish whether or not healthcare providers should harm their patients in pursuit of their own benefit). Delphi studies and other consensus methods use iteration paired with feedback to work toward a consensus among the expert panelists. Each cycle of collecting and analyzing information from the panel is called a round, and the length of a Delphi study is typically reported as the number of rounds. The goal of a Delphi study is to reach consensus, but how does one know when that has been achieved? Perfect agreement of experts is an unreasonable goal, so when does one stop? The conditions under which the study will conclude are critical to determine before data collection begins to avoid the researchers making arbitrary decisions during the study. The conditions should be reported in the method section of the resulting publication. This parallels the practice of setting hypotheses and the acceptable error rates prior to data collection rather than determining them post hoc in quantitative research.

The decision of how long the Delphi will continue is typically made in one of two ways: a priori or determined during the study by stopping criteria. An a priori number of rounds seems to be the more common approach (e.g., 71% of studies in one review; Diamond et al., 2014). When using this stopping condition, the predetermined number of rounds is conducted and the question asked of the final round of data from the panelists is “What degree of consensus was achieved?” (i.e., now that the study stopped, what is the consensus?).

Most recommendations for number of rounds for the Delphi state 2 or 3 as the preferred number, though there does not appear to be an empirical rationale for doing so (Boulkedid et al., 2011). Linstone and Turoff (1975) describe three rounds as typically sufficient to achieve stable results without overtaxing participants to the point where they are unhappy. This is consistent with two recent reviews that found 73% and 88% of Delphi studies finished with three rounds or less (Junger et al., 2017 and Foth et al., 2016, respectively). de Meyrick (2003) calls the appropriateness of this into question, however, as only 18 of the 126 Delphi studies he reviewed reported satisfactory results (i.e., strong degree of consensus) after the third round.

Those who do not take the a priori approach argue for a flexible number of rounds based on stopping criteria, which are typically based on a specified measure of consensus in panelist ratings. With this approach, the question of whether or not to stop is addressed after every round and is based on the degree of consensus already achieved (i.e., X level of consensus was observed, have criteria been met?). Practical considerations such as the likelihood of panelist dropout, panelist compensation funds running out, or time constraints should also be taken into consideration.

Ironically, there is no consensus on how to determine consensus in Delphi studies. One review of 126 studies found 33 different methods for identifying when consensus had been reached (de Meyrick, 2003). This is an important decision that hugely impacts the results. Grant et al. (2018) reanalyzed several Delphi data sets with 25 different consensus definitions found in the literature and found that depending on the method used, a range of 0%–84% of items reached consensus. Based on several reviews, the common criteria were percent agreement, mean or median ranking, lack of significant difference in ratings between rounds (e.g., Chi‐square, ANOVA, Wilcoxon signed‐rank test), proportion within a range, or decreased variance (Boulkedid et al., 2011; de Meyrick, 2003; Diamond et al., 2014). Kalaian and Kasim (2012) provide a nice overview of a number of methods along with concrete examples and formulas that can help you decide what to use. Only 45% of studies in Foth et al.'s (2016) review of nursing education literature reported setting consensus criteria a priori, though Junger et al.'s (2017) review focused on palliative care reported 88%.

Another consideration for when to end the Delphi process is stability (Nasa et al., 2021). The degree to which responses change between rounds (e.g., average rating for an item is 3.4 in Round 1 and 3.5 in Round 2) is considered even more important than the degree of consensus by some scholars (e.g., Chaffin & Talley, 1980). From this perspective the rationale is to stop the process when the results are stable, then assess the degree of consensus among responses. As with other decisions about when to stop the Delphi, the criteria for stability should be made a priori. These could be absolute standards (e.g., the average rating changes by no more than 0.2 points) or relative standards (e.g., the average rating changes by no more than 0.25 standard deviations). Nasa et al. (2021) argue for hierarchical stopping criteria based on assessment of the level of agreement and stability of responses.

### 2.3 How are data collected?

The next step is to design the survey that will be sent out to the expert panel. A classic Delphi will begin with open‐ended questions that ask the expert panel to contribute ideas toward the study's focus. The panelists are also typically given space to provide rationales for their suggestions, which may be included in future rounds (see below). When there is a body of literature available to inform the design of items for the consensus survey process, the initial open‐ended round is often omitted to save resources (both material and the panelists' time/energy). For example, in their Delphi to establish preliminary supervisor competencies for genetic counseling, Eubanks Higgins et al. (2013) used competencies from related fields to generate the items for the first survey. While this is fairly common practice (48% of studies in a Foth et al., 2016 review by Foth et al.), some scholars (e.g., Iqbal & Pipon‐Young, 2009; Rowe et al., 1991) highlight the potential for missing out on experts' ideas that are not in the literature and/or limiting experts' creativity. The ideas and experiences of practitioners are often most vulnerable to being overlooked by omitting the open‐ended round, though this can be somewhat mitigated by including an item in the consensus survey that asks for additional components or contributions that were not asked about.

Whichever way the initial items are generated, once those items exist, the next step is to have the expert panel start rating the items. The framing of the rating will depend on the research question and can take a variety of forms. One common item structure is “How X is Y to do Z?” Terms used for X are often “important,” “critical,” “useful,” “essential,” or similar quantifiable terms. The items that the researcher wants rated go in Position Y. Position Z is used to set the specific context of the question. For example, “How critical (X) is it to be able to assess genetic risk (Y) to provide genetic counseling (Z)?” Participants rate each item on a scale, with typical ranges being 1 to 4–10. Examples of anchors are “not at all X” for 1 and “completely X” for the highest score. Asking for a rationale for the rating is often helpful, but may not be feasible if there are a large number of items to rate. An alternative if there are a large number of items is to ask for explanations above or below a certain threshold (e.g., < 6).

### 2.4 What happens after participants provide ratings?

**FIGURE 2:**
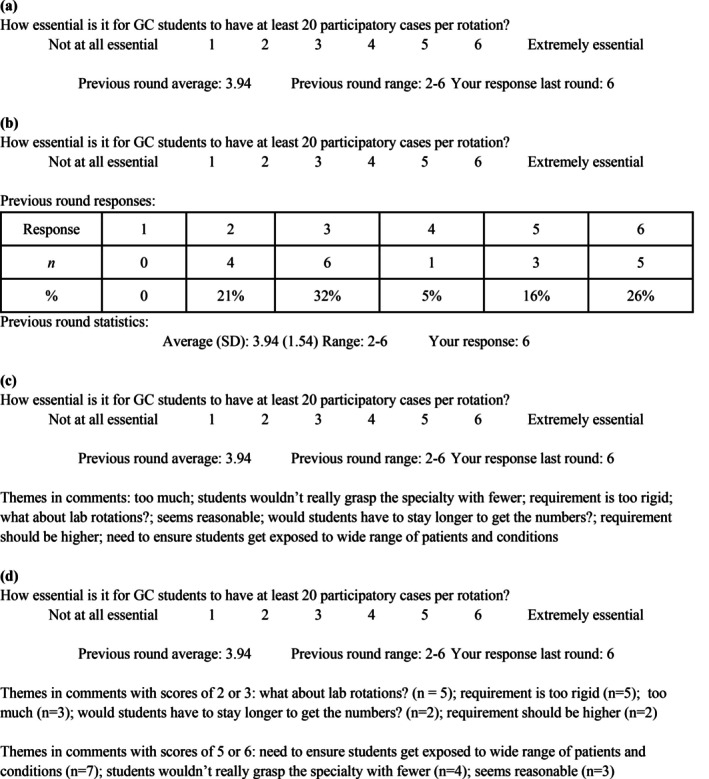
Examples of hypothetical Delphi survey items with various methods of feedback: (a) minimal quantitative feedback; (b) more extensive quantitative feedback; (c) minimal quantitative and qualitative feedback; and (d) minimal quantitative and extensive qualitative feedback.

One of the hallmark features of Delphi methods is the use of controlled feedback between rounds to help panelists reach consensus. The type of feedback varies across studies and can impact if and how consensus is reached. Feedback can be quantitative and/or qualitative. Quantitative feedback typically provides context for the degree of agreement of the panel as a whole for the statement being assessed (e.g., mean or median response) as well as the degree of agreement between panelists (e.g., range, standard deviation, or interquartile range; Humphrey‐Murto et al., 2017). Frequency tables and/or graphs are another common strategy for quantitative feedback. The panelist's response from the previous round is also frequently included because it may have been weeks since the last round of data collection took place. Qualitative feedback typically takes the form of comments written by panelists about items on the survey. These comments may be focused on rationales for high or low ratings, concerns about the phrasing of the item, or other aspects specific to the nature of the items included. Some researchers share all comments, while others only include comments from participants who rated the item particularly high or low relative to the rest of the panel. The researchers can provide the feedback as a separate document or integrate it into the next round of the survey (see Figure 2 for examples of how to do the latter). It may be beneficial to provide the feedback as part of the next survey to increase the likelihood of panelists reviewing it prior to completing the survey. Whether qualitative or quantitative, the feedback is controlled because it is filtered back through the research team, who decides what is shared with panelists in the next round.

How the feedback exerts its influence is not fully understood, as there can be multiple reasons that feedback shifts the perceptions of panelists. Rowe et al. (1991) applied the concept of informative versus normative influence of group dynamics from social psychology to Delphi methodology. Informative influence describes changes due to the persuasiveness of the rationale used by others. This is the type of influence researchers hope for, as it facilitates true consensus. Normative influence is the pressure to change to be aligned with or accepted by the group. Normative pressure is what the Delphi process is supposed to protect against by keeping the panelists separated, but Rowe et al. point out that when panelists are only provided numerical feedback (typically means or medians), there is not sufficient information about rationales to create informative influence and the changes are more likely due to normative pressure. There is also evidence that majority responses influence minority responses in Delphi studies even when the majority endorses an incorrect answer (Rowe et al., 2005), as has been well‐established in social psychology since Ashe's conformity studies (e.g., Ashe, 1956). Those with higher initial confidence in their expertise are also less likely to modify their responses in subsequent rounds due to feedback (Rowe et al., 2005). As the number of rounds increases, there is also a tendency for panelists to become less engaged and agree with the majority to bring the study to a close (de Meyrick, 2003).

The potential mechanisms by which feedback influences ratings are important to consider, especially as the reporting of feedback varies greatly across studies. Foth et al. (2016) found 55% of reviewed studies did not report whether feedback was provided to panelists, so it is difficult to assess potential normative influences in panelists' ratings. In Junger et al.'s (2017) review, 27% provided quantitative feedback only, 20% provided qualitative feedback only, and 13% provided both. It would seem prudent to include both quantitative and qualitative feedback to mitigate normative pressures, but doing so also increases the survey burden of the panelists and the amount of time the researchers need to analyze results before getting the next round delivered. Researchers should thoughtfully consider the manner of feedback that will be provided for participants and the impacts these choices may have on their results.

### 2.5 How should Delphi findings be reported?

There are no widely adopted reporting guidelines for Delphi methods like there are for qualitative methods (COREQ; Tong et al., 2007), genetic counseling intervention studies (GCRIS; Hooker et al., 2017), or clinical trials (CONSORT; Butcher et al., 2022; Schulz et al., 2010). Several authors have put forward recommendations for best practices in conducting and reporting Delphi studies in healthcare settings (e.g., Boulkedid et al., 2011; Diamond et al., 2014; Humphrey‐Murto et al., 2017). Junger et al.'s (2017) Recommendations for the Conducting and Reporting of Delphi Studies (CREDES) was an attempt to produce a more comprehensive set of guidelines that came out of the palliative care literature. More recently, two additional sets of reporting guidelines have been proposed. The DELPHISTAR guidelines are the product of a Delphi study of Delphi experts in social and health sciences and aim to provide an interdisciplinary standard (Niederberger et al., 2024). ACCORD, also developed using a modified Delphi method, is a broader set of guidelines meant for any consensus methodology in biomedical sciences (Gattrell et al., 2024). Given how new these guidelines are, it remains to be seen how widely they are adopted, but these resources can be a helpful place to start for those preparing manuscripts for publication. Due to the iterative nature of collecting and analyzing data, many authors find it helpful to write a combined method and results section to more clearly delineate how the results from one round influenced the next. Some journals are more amenable to this format than others, so it can be helpful to review how Delphi studies have been published in a targeted journal and/or asking the journal about formatting options prior to submission.

Given the flexible definitions of the Delphi approach, it is challenging to compare across studies due to methodological differences, especially as the lack of widely established reporting guidelines often means methodological details are omitted. Several systematic reviews have identified inconsistency in the reporting of Delphi studies (e.g., Foth et al., 2016; Junger et al., 2017). As discussed above, various subforms of Delphi methodology exist (e.g., policy Delphi) and it is critical to be specific about the design when using any form of Delphi methods. The term “modified Delphi” is frequently found in the literature. Some modifications may contradict the four core components of Delphi methods described above or change the methodology such that it is actually another consensus method (Boulkedid et al., 2011; Crisp et al., 1997). One example of the latter is the estimate, feedback, talk, estimate (EFTE) Delphi, otherwise known as interactive Delphi, where panelists meet together to complete surveys and discuss the results (Nelms & Porter, 1985). The authors describe this as a modified Delphi, although the design mirrors a nominal group technique method. Humphrey‐Murto et al. (2017) responded to a series of systematic reviews that found little methodological consistency across studies labeled as Delphi or modified Delphi by going so far as to recommend discontinuing the use of Delphi labels entirely, instead being thorough in describing the actual procedure. We do not endorse this position because the core features of a Delphi are widely agreed upon and if the terminology is used correctly it provides a significant amount of information concisely. Distinguishing between Delphi, normative group, and RAM methods is a useful framework but only if the terms are being used accurately. We strongly agree with the importance of being specific about study design elements and execution decisions, especially while there is inconsistency in consensus methodology terms. We hope guidelines like DELPHISTAR and ACCORD can help stabilize terminology to promote rigorous reporting of research.

### 2.6 Limitations of Delphi methods

Many of the limitations and critiques of Delphi methods come from a positivist framework and quantitative methodology. A positivist approach seeks to measure objective reality in a manner free from bias. The ability to assess the validity of the findings is the most important evaluation in a positivist approach. However, the literature lacks rigorous validity assessment of Delphi results (e.g., Humphrey‐Murto et al., 2017), partly because of the methodological inconsistencies discussed above (e.g., Foth et al., 2016; Junger et al., 2017). A deeper explanation for the challenge of establishing validity is that Delphi methods are typically used when there is insufficient data upon which to base a decision, which limits the ability to assess concurrent validity.

Concerns about validity can take several forms. Some critics of Delphi methods point to data showing it is not consistently more effective than other consensus methods and that the iterative process does not always produce better results than the first‐round ratings (e.g., Rowe et al., 1991). Others claim the researchers cannot help but mold panelists' opinions to some extent by selecting what type of feedback to provide (e.g., Hsu & Sandford, 2007). Grant et al. (2018) specifically call out the ability to shift conclusions dramatically based on the measure of consensus used. They argue that without preregistration of an analysis plan, it is impossible to know if the researchers were data mining. Preregistration of research designs and data analysis plans is increasingly being called for in science to help distinguish between hypotheses at the outset of the study and using the data collected to create an explanatory framework for observed results (e.g., Hardwicke & Wagenmakers, 2023; Munafò et al., 2017; Nosek et al., 2018), though not all scientists agree with the idea (e.g., Garzino Demo, 2025; Pham & Oh, 2021). These plans are published in online databases such as the Open Science Framework, Prospero, and the Center for Open Science, and hold researchers accountable for deviations in protocol.

The issue of validity of results should not be set aside lightly, but there is an opportunity to conceptualize consensus methods broadly and Delphi methods specifically from other paradigms. Junger et al. (2017) describe Delphi studies as “a heuristic device that relies on expert knowledge to negotiate a shared reality and to co‐construct knowledge, rules, and recommendations” (p. 701). This description suggests a constructivist framework, more common in qualitative research, may be more appropriate. To determine if the outputs of the Delphi produce intended results (e.g., better health outcomes, reduction in patient wait times, improved self‐efficacy); however, additional research is needed. Similar to the utility of qualitative methods to identify patterns and experiences that need to be explored quantitatively to assess generalizability, the Delphi method benefits from follow‐up research to test whether the expert opinions prove accurate. For example, the proposed supervision competencies outlined by Eubanks Higgins et al. (2013) provided a reference for important supervisor knowledge and skills. The issue is there has been insufficient follow‐up research to determine if these competencies actually impacted supervision, training, or patient care outcomes. Without data to support the efficacy of the competencies, it is unclear to what extent they should be implemented in any systematic manner. Researchers should thoughtfully consider the philosophical framework (e.g., positivist, constructivist, pragmatic) with which they are going to approach Delphi research and the way that framework influences choices such as how to assess the rigor of the study both before beginning and in the discussion section of their article.

## 3 SUMMARY

Consensus methodology and Delphi studies are valuable in genetic counseling research, particularly when empirical evidence is limited or evolving. Their structured, iterative design enables the integration of expert perspectives across a range of applications, including guideline development, educational curriculum design, competency definition, quality assessment, and outcome standardization. This article not only highlights the breadth of Delphi applications in genetic counseling but also provides practical guidance on key methodological decisions—such as expert panel selection, number of rounds, survey design, and reporting standards. By thoughtfully considering and addressing these methodological choices, researchers can enhance the rigor and impact of their studies. We encourage genetic counseling researchers to consider incorporating Delphi methods into their work, and when appropriate, to collaborate with methodologists or statisticians with expertise in consensus techniques to strengthen study design, implementation, and impact of research.

## AUTHOR CONTRIBUTIONS

I.M. and H.Z. contributed to the conceptualization, drafting and editing of the manuscript.

## FUNDING INFORMATION

No funding was provided for this article submission.

## CONFLICT OF INTEREST STATEMENT

Dr. Heather Zierhut is a member of the editorial board for the *Journal of Genetic Counseling* and co‐author of this article. They were excluded from editorial decision‐making related to the acceptance of this article for publication in the journal. All authors (I.M.M. and H.A.Z.) declare that they have no conflicts of interest to disclose.

## ETHICS STATEMENT

Human Studies and Informed Consent: Statement is not applicable for this submission.

Animal Studies: Statement is not applicable for this submission.

## Data Availability

No original data is presented in this submission.
